# Novel Nanobiocomposites Based on Natural Polysaccharides as Universal Trophic Low-Dose Micronutrients

**DOI:** 10.3390/ijms222112006

**Published:** 2021-11-05

**Authors:** Spartak S. Khutsishvili, Alla I. Perfileva, Olga A. Nozhkina, Tatjana V. Ganenko, Konstantin V. Krutovsky

**Affiliations:** 1 Department of Physical Organic Chemistry, N.N. Vorozhtsov Novosibirsk Institute of Organic Chemistry, Siberian Branch of the Russian Academy of Sciences, 9 Lavrentiev Av., 630090 Novosibirsk, Russia; khutsishvili_sp@yahoo.com; 2 Laboratory of Plant-Microbe Interactions, Siberian Institute of Plant Physiology and Biochemistry, Siberian Branch of the Russian Academy of Sciences, 664033 Irkutsk, Russia; alla.light@mail.ru (A.I.P.); smallolga@mail.ru (O.A.N.); 3 Laboratory of Functional Nanomaterials, A.E. Favorsky Irkutsk Institute of Chemistry, Siberian Branch of the Russian Academy of Sciences, 1 Favorsky Str., 664033 Irkutsk, Russia; ganenko@irioch.irk.ru; 4 Department of Forest Genetics and Forest Tree Breeding, Faculty of Forest Sciences and Forest Ecology, Georg-August University of Göttingen, Büsgenweg 2, 37077 Göttingen, Germany; 5 Center for Integrated Breeding Research (CiBreed), Georg-August University of Göttingen, Albrecht-Thaer-Weg 3, 37075 Göttingen, Germany; 6 Laboratory of Population Genetics, N.I. Vavilov Institute of General Genetics, Russian Academy of Sciences, Gubkin Str. 3, 119333 Moscow, Russia; 7 Genome Research and Education Center, Laboratory of Forest Genomics, Department of Genomics and Bioinformatics, Institute of Fundamental Biology and Biotechnology, Siberian Federal University, 660036 Krasnoyarsk, Russia; 8 Forestry Faculty, G.F. Morozov Voronezh State University of Forestry and Technologies, 8 Timiryazeva Str., 394036 Voronezh, Russia

**Keywords:** bacteria, *Clavibacter sepedonicus*, in vitro, manganese, nanobiocomposite, natural polysaccharides, phytopathogens, potato, rhizosphere, *Solanum tuberosum* L.

## Abstract

New promising manganese-containing nanobiocomposites (NCs) based on natural polysaccharides, arabinogalactan (AG), arabinogalactan sulfate (AGS), and *κ*-carrageenan (*κ*-CG) were studied to develop novel multi-purpose trophic low-dose organomineral fertilizers. The general toxicological effects of manganese (Mn) on the vegetation of potatoes (*Solanum tuberosum* L.) was evaluated in this study. The essential physicochemical properties of this trace element in plant tissues, such as its elemental analysis and its spectroscopic parameters in electron paramagnetic resonance (EPR), were determined. Potato plants grown in an NC-containing medium demonstrated better biometric parameters than in the control medium, and no Mn accumulated in plant tissues. In addition, the synthesized NCs demonstrated a pronounced antibacterial effect against the phytopathogenic bacterium *Clavibacter sepedonicus* (*Cms*) and were proved to be safe for natural soil microflora.

## 1. Introduction

Despite the wide application of modern trace element-containing fertilizers, agriculture faces strong challenges associated with a need for optimal plant nutrition during the growing season. To address these challenges, researchers have great hopes for nano-sized materials [1,2,3,4,5]. Nowadays, taking into account the growing demand of the world market, great efforts are being directed towards the development of a new generation of complex mineral fertilizers. Such fertilizers should improve plant uptake of trace nutrients in safe doses, ensure their delayed effect, and be resistant to the leaching of microelements from different types of soil. These fertilizers will permit a larger harvest, and the better uptake of nutrients minimizes their loss in the environment. In addition, every year the search for more efficient and safer fertilizers becomes more and more pressing, especially in the context of climate change and problems of healthy nutrition. At present, nanobiocomposite (NC) materials are extensively used in various fields of biology and agricultural production, in particular, in the development of organomineral fertilizers containing highly effective trace element additives [2,6,7,8,9,10]. Modern composite biomaterials incorporating metal nanoparticles possess increased resistance to external actions, can change solubility, may acquire a higher biological activity, etc. [11,12,13,14]. Biopolymer materials are very prospective and efficient stabilizing matrices of nano-sized metal particles [15,16]. They are capable of forming advanced functional materials, including those needed for the production of fertilizers derived from metal-containing NCs. However, the global community imposes stringent requirements on research methods and controls the use of nanosubstances for agriculture [17]. Undoubtedly, the safest and most environmentally friendly approach is based on the application of various natural compounds for the synthesis of NCs, which contain reducing and stabilizing fragments [18,19,20]. At the same time, nanochemistry also faces such issues as the design of biocompatible, safe, and easily biodegradable nanosubstances. Therefore, the employment of natural polysaccharides as matrices is quite justified [6,21,22].

In the present work, special attention was paid to the effect of various concentrations of Mn in plants, as Mn plays an important role in the biochemical processes occurring in vegetables [23]. Using the model system of potatoes (*Solanum tuberosum* L.) in vitro, we have studied the qualitative and quantitative influence of Mn and Mn-containing NCs on plant growth, obtained additional information on the migration of Mn ions, evaluated the general toxicological effect of this metal on the development of the plants, and found optimal conditions for Mn intake into the plants using *S. tuberosum* L. as an example. It is known that the lack or excess of Mn can lead to diseases and low yields of potatoes [24]. Mn is a cofactor of many plant cell enzymes involved in redox reactions of photosynthesis (photoproduction of oxygen in chloroplasts); the synthesis of vitamins *C*, *B*, *E*; and ascorbic acid [25]. In addition, Mn increases the content of sugars and their outflow from leaves, and accelerates plant growth and seed ripening. A deficiency of Mn impedes the synthesis of organic substances, decreases chlorophyll content, and induces point leaf chlorosis [26,27]. At the same time, the accumulation and excess of Mn also have negative effects, which affect both the state of the plant and the health of humans consuming such plants. The in-depth study of metabolism occurring in potatoes (one of the most important agricultural crops, *S. tuberosum* L.) and its optimal nutrition is of great interest to biochemists and plant physiologists.

In this study, novel Mn-containing NCs based on natural polysaccharides (AG, AGS, and *κ*-CG) have been synthesized to design new multi-purpose trophic low-dose micronutrients. Biopolymer matrices were employed earlier as gelling and binding agents, components for tissue engineering, and carriers for target therapy, and were capable of forming stable composite materials [21,22,28,29]. It should be noted that potatoes are quite sensitive to the concentration of Mn [30]. Therefore, we needed to determine what content of this element in the NCs and in the culture medium of potato *Solanum tuberosum* L. would be optimal for the development of plants based on the obtained physicochemical characteristics and biometric parameters, and to evaluate the protective properties of Mn-containing NCs against phytopathogens and their safety for soil microflora. The results obtained by biological experiments, EPR, and other physicochemical methods open up new possibilities in the development of universal safe NCs for plant protection and the targeted delivery of trace elements.

## 2. Results

### 2.1. Toxic Effect of Mn and Its Accumulation during the Growing Season of Solanum tuberosum

The concentration of Mn in the plant growth medium varied from the complete absence of Mn sulfate in the nutrient medium to a 100-fold excess over the control (Table 1). It should be noted that Mn is considered an extremely immobile element, and in plants it only moves upward through the xylem to the leaves. After reaching the leaves, Mn is not transferred to other parts of the plant [25]. Indeed, the highest accumulation of Mn was observed in the green photosynthetic part of *S. tuberosum* L., including the leaves.

**Figure 1:**
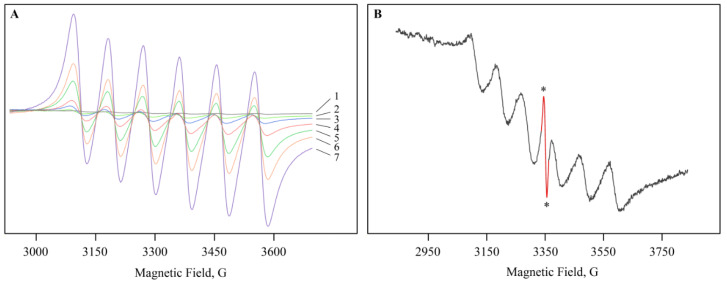
The EPR spectra in leaves with (**A**) and without (**B**) Mn sulfate in the nutrient medium. (**A**): (1)—0 mol/L, (2)—0.1 mol/L (control), (3)—0.2 mol/L, (4)—0.5 mol/L, (5)—1 mol/L, (6)—2 mol/L, (7)—10 mol/L. (**B**): the additional line is indicated by the asterisks.

Mn ions were easily monitored, since the ground state of high-spin complexes with *d*^5^ configuration is an orbital singlet, and the EPR spectrum contained six lines of hyperfine structure (HFS) from Mn^2+^ with spin 5/2 [31]. The multiplet, observed in the spectra of roots, stems, and leaves, had the following spectral characteristics: a g-factor in the region of 2.004(2), a constant (*A*) 95(2) G, and a line width (Δ*H*) 38(5) G; see Figure 1. A characteristic signal with a structure is typical for freely rotating Mn ions in the octahedral oxide environment of the Mn(H_2_O)_6_^2+^ complex and is very often detected in various biological materials of plants [32]. The EPR study of plant tissues evidenced that the intensity of the sextet increased (by approximately 70–75 times) with a growth of Mn sulfate concentration in the nutrient medium by up to 10 mol/L (Figure 1A). At the same time, the width and shape of the lines did not noticeably change with a higher content of salt (0–10 mol/L). The concentration of Mn^2+^ ions in plant organs was insufficient for significant broadening of lines, due to dipole–dipole interactions, and some alteration of the line shape was explained by inhomogeneous broadening [33].

Note that the EPR spectra of plant tissues also showed a singlet with a *g*-factor of 2.004(2) and a line width (Δ*H*) 8(1) G (Figure 1B). The recorded signal did not change intensity in the sample; therefore, it can be assigned to long-lived radicals. The signal parameters are characteristic of stable semiquinone radicals and are found in many plant materials [32,34]. The level of semiquinone radicals can serve as an indicator of a plant’s resistance to stress and is determined by the degree of damage to the cell structure, moisture loss, and other processes.

**Figure 2:**
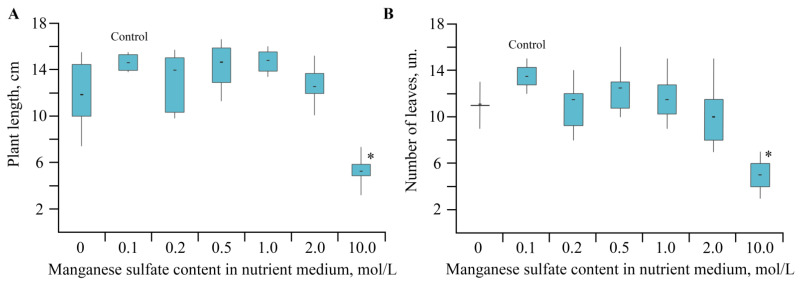
(**A**,**B**) The biometric parameters depending on Mn sulfate concentration in nutrient medium; * *p* ≤ 0.01 compared to the control according to the Mann–Whitney test.

**Figure 3:**
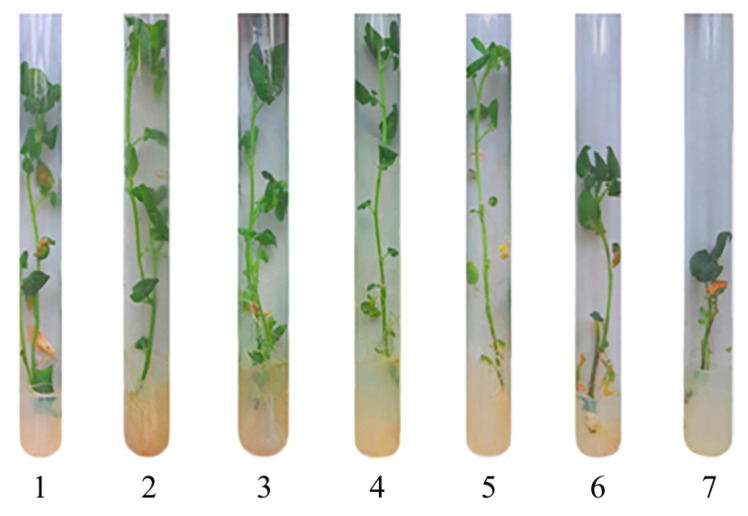
Photos of potatoes grown with different Mn sulfate content in the nutrient medium; (1)—0 mol/L, (2)—0.1 mol/L (control), (3)—0.2 mol/L, (4)—0.5 mol/L, (5)—1, (6)—2 mol/L, (7)—10 mol/L.

The change in concentration of Mn sulfate (0–10 mol/L) in a nutrient medium had a pronounced effect on plant growth and plant biometric parameters (Figure 2 and Figure 3).

### 2.2. Structural Features of Mn-Containing NCs

**Figure 4:**
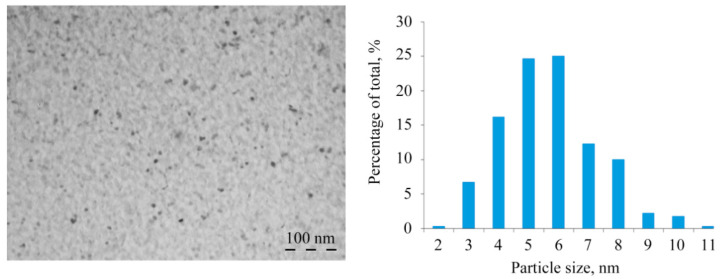
Typical TEM micrograph and size distributions of nanoparticles of Mn-containing NCs for *κ*-CG-Mn.

Natural polysaccharides formed aggregately stable NCs (AG-Mn, AGS-Mn, and *κ*-CG-Mn), in which Mn-containing nanoparticles were stabilized by a biopolymer shell. According to TEM data, in the films of the obtained NCs in the matrix, electron-dense round nanoparticles with an average size of 3–6 nm were generated (Figure 4). Nanoparticles formed almost no cluster groups and were usually distant from each other in the bulk of the polymer.

**Figure 5:**
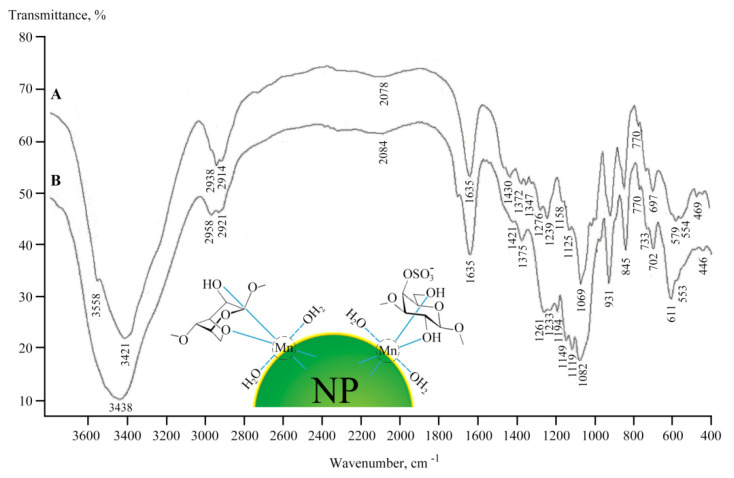
FTIR spectra of original polysaccharide *κ*-CG (**A**) and NC *κ*-CG-Mn (**B**).

The nanoparticles were most likely formed as Mn(OH)_2_×nH_2_O and were stabilized in the matrix on oxygen atoms and hydroxyl groups [35,36] (Figure 5). Polysaccharides have a propensity to form corresponding coordination biopolymer complexes with some ions of mono and divalent metals, which can be located on the surface of metal-containing nanoparticles. According to data from the Fourier transform infrared (FTIR) spectroscopy, the formation of these NCs was not accompanied by structural changes in the polysaccharide (Figure 5). Comparative analysis of the FTIR spectra of the initial polymers and NCs revealed a broadening of the absorption bands in the high frequency region of the FTIR spectra of NCs at 3200–3600 cm^−1^, which is attributable to the stretching vibrations of the hydroxyl groups *δ*(OH), thus indicating complexation with Mn^2+^ and the presence of bound water in the compounds [37,38,39]. The broadening of OH stretching vibrations during complexation is associated with the participation of sugar OH groups in metal–ligand bonding. It should be noted that the main broadenings of OH stretching vibrations in the spectra of the NCs were due to the presence of a strong hydrogen network between the OH groups of the polysaccharide matrix and H_2_O molecules in the metal–polysaccharide crystal structures, which are responsible for the stabilization of solid structure and for retaining aggregated units. Such a system of hydrogen bonding was found in the crystal structure of Mn(D-gluconate)_2_×2H_2_O salt [40]. In addition, the maximum of the absorption band from *δ*(OH) vibrations (at 1430 cm^−1^), characteristic of one of the possible positions of the CH_2_OH group, shifted towards lower frequencies, which also evidences ionization due to complexation [39].

**Figure 6:**
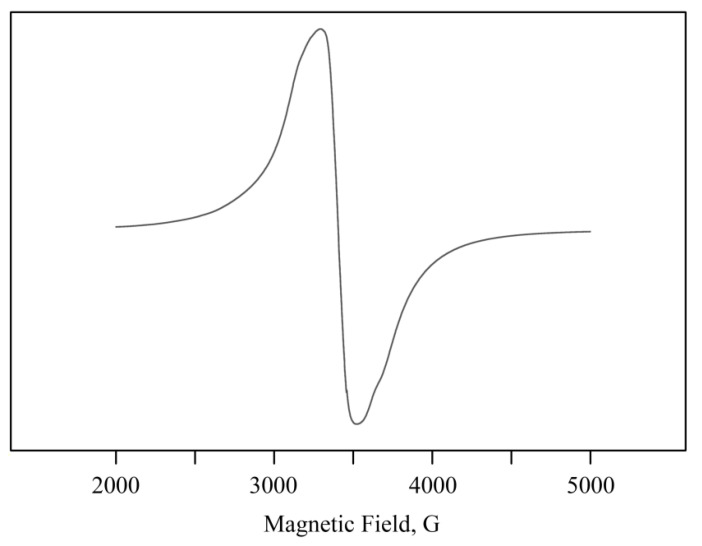
Typical EPR spectrum of Mn-containing NCs for *κ*-CG-Mn.

NCs are paramagnetic. Their EPR spectra showed intense broad complex lines or isotropic Lorentzian lines (in almost ideal cubic symmetry [41]) with an average effective *g*-factor of 2.07(1) and a width (Δ*H*) of 250–450 G. The narrowing of the line width (Δ*H*) of the Mn^2+^ signal from about 500–600 G peak-to-peak to 200–250 G could be attributed to the narrowing of the exchange [42,43] (Figure 6). The close proximity of Mn^2+^ ions could have caused strong dipolar and spin exchange interactions that may have resulted in a single-line EPR spectrum with a high concentration of Mn in the sample. As the content of Mn^2+^ increased, Mn-containing clusters were generated. This gradually degraded the resolution of the HFS components in the spectrum [44], which was consistent with the TEM data.

### 2.3. The Effect of Mn-Containing NCs on the Growth and Development of Solanum tuberosum L.

**Figure 7:**
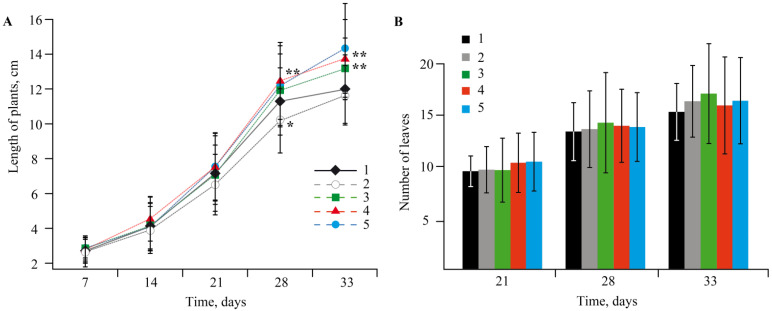
Dynamics of the biometric parameters of length of plants (**A**) and number of leaves (**B**) in the control medium and in plants grown in a nutrient medium with NCs; (1)—control, (2)—without Mn sulfate in medium, (3)—AG-Mn, (4)—AGS-Mn, (5)—*κ*-CG-Mn; * *p* ≤ 0.05 and ** *p* ≤ 0.01 compared to the control according to the Mann–Whitney test.

**Figure 8:**
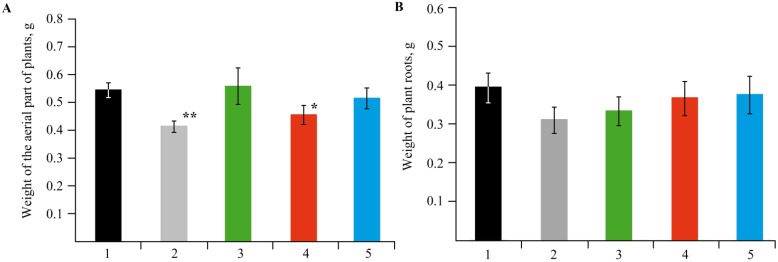
Mass of the vegetation of plants (**A**) and the biomass of roots (**B**) in the control medium and in plants grown in a nutrient medium with NCs; (1)—control, (2)—without Mn sulfate in medium, (3)—AG-Mn, (4)—AGS-Mn, (5)—*κ*-CG-Mn; * *p* ≤ 0.05 and ** *p* ≤ 0.01 compared to the control according to the Mann–Whitney test.

The experiments showed that NCs had no negative effect on potato growth and development; all the plants were bright green, and their biometric parameters were similar to the control plants (Figure 7 and Figure 8).

After 7 days of growing, differences in the formation of root systems and the color of the aboveground parts were visually observed in the plants. For instance, in the control group, single roots began to appear in 40% of the plants (from 2 to 7 thin roots per plant). The presence of AG-Mn somewhat reduced intensity of root formation; only 25% of the plants formed a root system (from 1 to 8 very small roots per plant). In the medium with AGS-Mn, 50% of the plants had already formed roots (from 2 to 5 thick roots per plant). In case of *κ*-CG-Mn, 60% of the plants had already a root system, while the roots were thick and vigorous (from 1 to 5 roots per plant).

After 14 days, the control group had many long thin roots. In most cases, the root system was well developed, and aerial roots were detected. Yellow leaves were observed in only a few cases. The plants grown in a medium with AG-Mn did not differ from the control ones in terms of the root system; the color of most plants was deep green. When potatoes were cultivated in an AGS-Mn-containing medium, the plants were rich bright green and their leaves were large. Each plant had either many long thin roots or several long thick roots. They looked the most viable of all the studied samples. At the same time, the stimulating effect of *κ*-CG-Mn, which was observed in the first week of growing potatoes, leveled out; some of the plants had yellowed leaves and their root systems was represented by 2–5 long thin roots.

On the 21st day of incubation, in the control group, the root systems of the plants were extensively developed; one third of the plants had single yellowed leaves. The addition of AG-Mn to the potato growth medium resulted in a moderately developed root system in the plants, calluses were observed rarely, and 15% of the plants had yellowed leaves. The most viable state was detected in the potatoes grown in the medium with the addition of AGS-Mn; the color of the aboveground organs was brighter green than in the group with AG-Mn, the root system was extensively developed, and yellowed leaves were noted in only 10% of the plants. Potatoes grown in a nutrient medium with *κ*-CG-Mn showed a strong root system, while 45% of the plants had yellowed leaves, and a few plants were pale green.

After 28 days of incubation, the root systems of the control group were often well developed, calluses were detected in a few cases, and dead and yellowish leaves appeared. The state of the plants grown in media with AG-Mn and AGS-Mn was similar to their state after 21 days of incubation. The plants with *κ*-CG-Mn turned out to be the most viable. They had a bright green color, a well-developed root system, the number of dead and yellowed leaves was less in comparison with the control, and no calluses was observed.

The EPR studies showed that in all plant tissues (roots, stems, and leaves) grown in a medium with Mn-containing NCs, as well as in the control group, a characteristic multiplet from Mn^2+^ ions was registered (*g*-factor in the region of 2.0031(2), constant (*A*) 94(2) G of HFS). A comparison of Mn content in plant organs was also made with the control (Table 2).

### 2.4. Effect of NCs on the Stress Resistance of Potato Plants

To evaluate the effect of the obtained NCs on the stress resistance of potato plants, the following biochemical parameters were measured: peroxidase activity in the tissues of potato leaves; the content of reactive oxygen species (ROS); and the amount of lipid peroxidation (LPO) products, diene conjugates (DC), and malonic dialdehyde (MDA) in roots and leaves.

**Figure 9:**
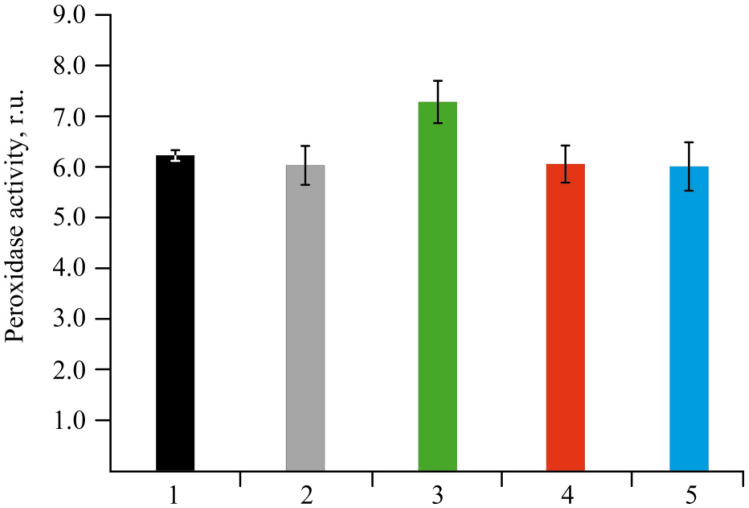
Effect of NC treatments on the peroxidase activity in potato plants in comparison with the control group in vitro; (1)—control, (2)—without Mn sulfate in medium, (3)—AG-Mn, (4)—AGS-Mn, (5)—*κ*-CG-Mn. The treated plants were not different from the control according to the Mann–Whitney test.

Peroxidase is a very sensitive enzyme to various stress factors. Therefore, changes in the activity of this enzyme can often be used to assess the body’s resistance to stress [45,46]. The activity of guaiacol-dependent peroxidase was determined by the Boyarkin method at the end of the plants’ co-incubation with NCs [47] (Figure 9).

**Figure 10:**
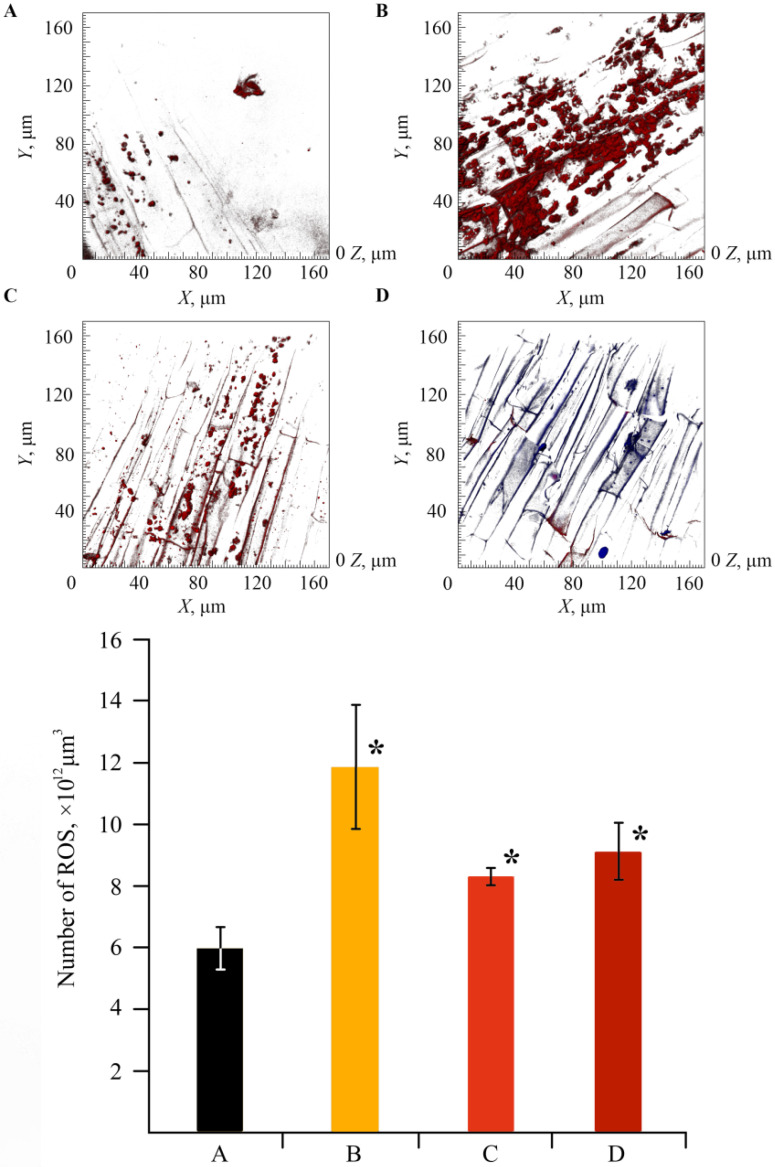
Confocal laser scanning microscopy (CLSM) images of the effect of NCs on ROS production and respective data on the number of ROS in plant tissues in the control (**A**), infected by *Cms* (**B**), treated with NCs (**C**), treated with NCs and infected by *Cms* (**D**); * *p* ≤ 0.01 compared to the control according to the Mann–Whitney test.

Plants suffering from ring rot were used to assess the level of stress using ROS. They were completely colonized by the pathogen after four days had passed since the moment of their infection, since after this period of time, no increase in ROS was recorded in the roots (Figure 10B). The observed data are quite understandable, since it is known that the ROS level rises significantly at the initial stage of a plant’s response to a stress factor [48,49]. If healthy plants were treated with NCs, an increase in the amount of ROS in the potato roots by more than four times was observed as compared with the control (Figure 10C). This indicates an increase in the protective reaction of plant cells to stress due to the influence of NCs. In the case of NC treatment of plants with ring rot, a decrease in the ROS content was also noted (Figure 10D). The obtained result indicates the activation of the protective functions of the plant organism under stress.

Under any stress, plant cells produce ROS, messenger molecules that trigger a cascade of protective programs in a cell and are capable of destroying the cell itself and its structures, thus causing LPO. Therefore, to evaluate the presence of stress in plants under infection conditions, after treatment with NCs along and together with infection during the first stage of biochemical studies, we studied the ROS content in the potato roots.

**Figure 11:**
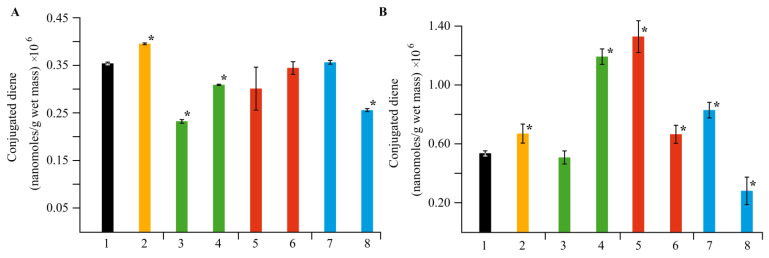
Effect of NC treatments on the content of DCs in potato leaves (**A**) and roots (**B**), in comparison with the control group in vitro, as well as in plants infected by *Cms* pathogen; (1)—control, (2)—plants infected by *Cms*, (3)—AG-Mn without *Cms*, (4)—infected plants in the presence of AG-Mn, (5)—AGS-Mn without *Cms*, (6)—infected plants in the presence of AGS-Mn, (7)—*κ*-CG-Mn without *Cms*, (8)—infected plants in the presence of *κ*-CG-Mn; * *p* ≤ 0.01 compared to the control according to the Mann–Whitney test.

During the first stage, the products of ROS damaging action on living plant cells are DCs. Therefore, a series of experiments was carried out to assess the amount of DCs in the potato roots and leaves under the studied stress factors (Figure 11). It was found that infection of potatoes with *Cms* increased the content of DCs and LPO intermediates, both in the roots and leaves, as compared to the control. In the case of *κ*-CG-Mn, the amount of DCs in the roots increased, while this parameter in the leaves remained at the control level. The addition of *κ*-CG-Mn to infected plants reduced the content of DCs in both roots and leaves, which indicated the inhibition of LPO processes. Treatment of healthy plants with AGS-Mn did not affect the DC level. The addition of this composite to infected plants decreased the content of DCs in roots and leaves as compared to the infected plants without NC treatment. This effect was most pronounced in samples extracted from plant roots. It was found that AG-Mn increased the content of DCs in the roots by two times compared to the control, while the opposite effect was observed in the leaves, where a decrease in DCs was observed. The addition of this composite to infected plants significantly increased the amount of DCs in the roots and decreased it in the leaves, as compared to infected plants without NC treatment.

**Figure 12:**
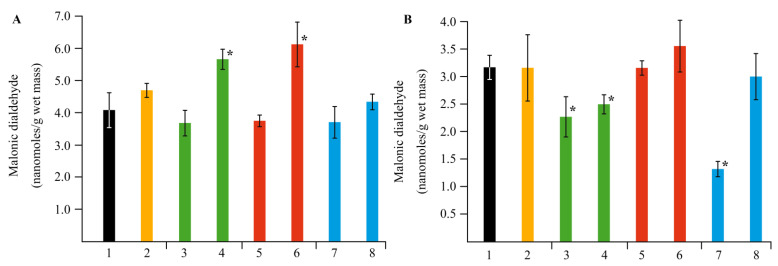
Effect of NC treatments on the content of malonic dialdehyde (MDA) in potato leaves (**A**) and roots (**B**), in comparison with the control group in vitro, as well as in plants infected by *Cms* pathogen; (1)—control without NCs and *Cms*, (2)—plants infected by *Cms* without NCs, (3)—AG-Mn without *Cms*, (4)—infected plants in the presence of AG-Mn, (5)—AGS-Mn without *Cms*, (6)—infected plants in the presence of AGS-Mn, (7)—*κ*-CG-Mn without *Cms*, (8)—infected plants in the presence of *κ*-CG-Mn; * *p* ≤ 0.01 compared to the control according to the Mann–Whitney test.

If the stress load on a plant organism is continuous or increases, MDA is formed in cells [50]. Therefore, during the next stage of our research, we studied MDA content in potato tissues (Figure 12). It was found that in plants infected with the *Cms* pathogen, the MDA content increased in both roots and leaves. Treatment of pathogen-free plants with *κ*-CG-Mn composite significantly reduced the content of MDA in both root and leaf tissues. Treatment of infected plants with *κ*-CG-Mn brought the MDA level in both roots and leaves to the control level. AGS-Mn had no effect on the MDA content in both roots and leaves. However, in infected plants, it significantly increased the amount of MDA in leaves. AG-Mn markedly reduced the MDA content in roots of both healthy and infected potato plants. AG-Mn increased the MDA content in leaves of the infected plants.

### 2.5. Antibacterial Activity of the NCs against the Clavibacter sepedonicus Phytopathogen

It is important to note that polysaccharide-based biopolymer composites can exhibit biological activity in plant protection [51,52,53]. In this regard, we decided to evaluate the antibacterial effect of the synthesized NCs. To reach this goal, a series of experiments was carried out to check the effect of NCs on the viability of *Cms,* the pathogen which causes potato ring rot. In most countries in the world, this bacterium is considered a quarantine species that causes serious crop losses of up to 50% [54,55]. The infection is latent and manifests itself as wilt and yellowing of the stems during growing season. It can only be revealed in the form of ring, when the tuber is cut longitudinally, while visually the tuber (seed material) may look absolutely normal. The problem is accentuated by the lack of efficient methods to combat this bacterium. All measures are only preventive and comprise the treatment of equipment and manual removal of infected plants from the fields. Therefore, the search for an agent to control this pathogen is an urgent necessity.

**Figure 13:**
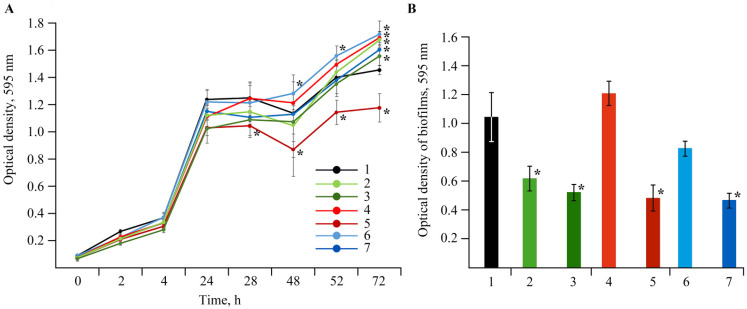
Effect of NC treatments with concentrations of Mn 0.000625% and 0.00625%^†^ in volume on growth (**A**) and biofilm formation (**B**) of *Cms* pathogen; (1)—control, (2)—AG-Mn, (3)—AG-Mn^†^, (4)—AGS-Mn, (5)—AGS-Mn^†^, (6)—*κ*-CG-Mn, (7)—*κ*-CG-Mn^†^; * *p* ≤ 0.01 compared to the control according to the Mann–Whitney test.

The experiments showed (Figure 13) that during 28 h, all NCs reduced bacterial growth. The bacteriostatic effect was further observed upon the introduction of composites with a concentration of 0.00625% Mn in volume. AGS-Mn with a Mn concentration of 0.00625% exhibited the most pronounced negative effect on bacterial growth. The bactericidal effect of the composites with respect to *Cms* was studied by the circle method (well method, agar diffusion method). It was revealed that AGS-Mn had such an effect. The zone of inhibited bacterial growth around the well reached 9.0 ± 0.3 mm. No pronounced inhibition of bacterial growth was found with AG-Mn and *κ*-CG-Mn.

**Figure 14:**
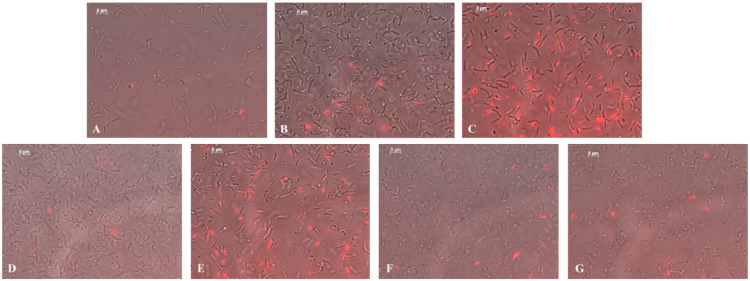
Micrographs of *Cms* biofilms in control (**A**) and in the presence of NCs (with concentrations of Mn 0.000625% and 0.00625%^†^ in volume for AG-Mn (**B**), AG-Mn^†^ (**C**), AGS-Mn (**D**), AGS-Mn^†^ (**E**), *κ*-CG-Mn (**F**), *κ*-CG-Mn^†^ (**G**).

The high survival rate of bacteria is mainly ensured by their ability to form biofilm [56]. Bacteria release special substances in which their cells are immersed that make them more resistant to any external actions [57]. Biofilm formation is also observed for ring rot. These bacteria are able to accumulate in the conductive channels of the plant causing their blockage and, hence, the wilting of the stem [58]. Therefore, we next studied the effect of NCs on biofilm formation of the potato ring rot pathogen. It was found that AG-Mn and AGS-Mn with a concentration of Mn 0.00625% in volume inhibited bacterial biofilm formation, with the percentage of dead cells reaching up to almost 25% (Table 3). In the control sample, the number of dead cells was very low (Figure 14A; Table 3). Treatment of the bacteria with NCs at a concentration of 0.000625% led to the appearance of dead cells (Figure 14B,D,F; Table 3). At a 10-fold higher concentration of the composites, the number of dead bacterial cells greatly increased (Figure 14C,E,G; Table 3). The maximum percentage of dead cells was detected in the case of AGS-Mn (~25%) and *κ*-CG-Mn (~14%) at concentrations of 0.00625% (Table 3). After incubation, the cells also thickened and shortened compared to the control (Table 3).

**Figure 15:**
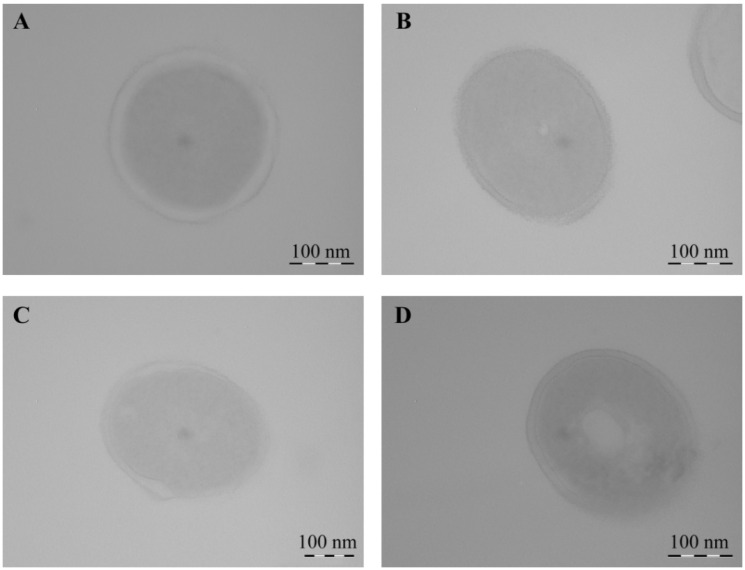
TEM micrographs of *Cms* bacteria in the control (**A**) and treated with AG-Mn (**B**), AGS-Mn (**C**), and *κ*-CG-Mn (**D**). Transverse optical section of cells.

**Figure 16:**
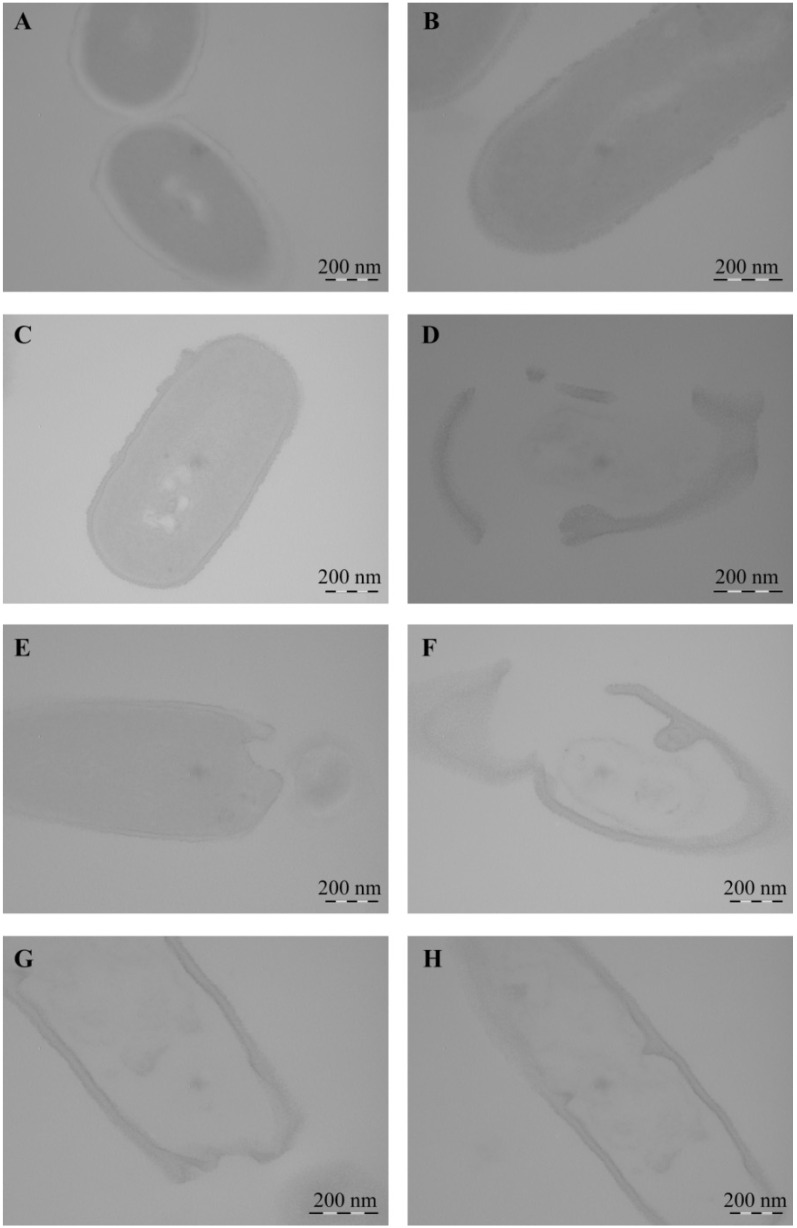
TEM micrographs of *Cms* bacteria in the control (**A**,**B**) and treated with AG-Mn (**C**,**D**), AGS-Mn (**E**,**F**), and *κ*-CG-Mn (**G**,**H**). Longitudinal optical section of cells.

Using TEM, it was shown that *Cms* bacteria in the control experiment, both in transverse (Figure 15) and longitudinal (Figure 16) optical sections, had thick, integral undamaged cell walls. After the incubation of bacteria with NCs for 1 h, the bacteria lost their native shape, the cell surface became uneven, and Mn-containing nanoparticles were not detected. A pronounced lysis of the cell wall was observed during the treatment of bacteria with AG-Mn and AGS-Mn, NCs and often only fragments of their cell wall were registered instead of bacteria (Figure 16). Treatment of *Cms* with κ-CG-Mn NC resulted in invaginations of the cell wall inside the bacterial cell (Figure 16G,H); no adhesion of several bacterial cells occurred. The obtained micrographs indicated the loss of turgor and the release of the cell contents.

### 2.6. Bactericidal Effect of NCs on Soil Bacteria

Today, pesticides are widely used everywhere, which is becoming a worrisome problem. While pesticides are sprayed directly at plants and soil, only about 1% of the sprayed pesticide reaches the target [59]. Pesticides accumulate in the environment, polluting its water and soil, and having a negative impact on its inhabitants [60,61,62]. In this regard, it is extremely important to search for environmentally benign substances to improve the state of the environment and of cultivated plants. Therefore, an important question regarding new Mn-containing NCs is their impact on the environment, in particular, on the viability of the soil microbiome.

**Figure 17:**
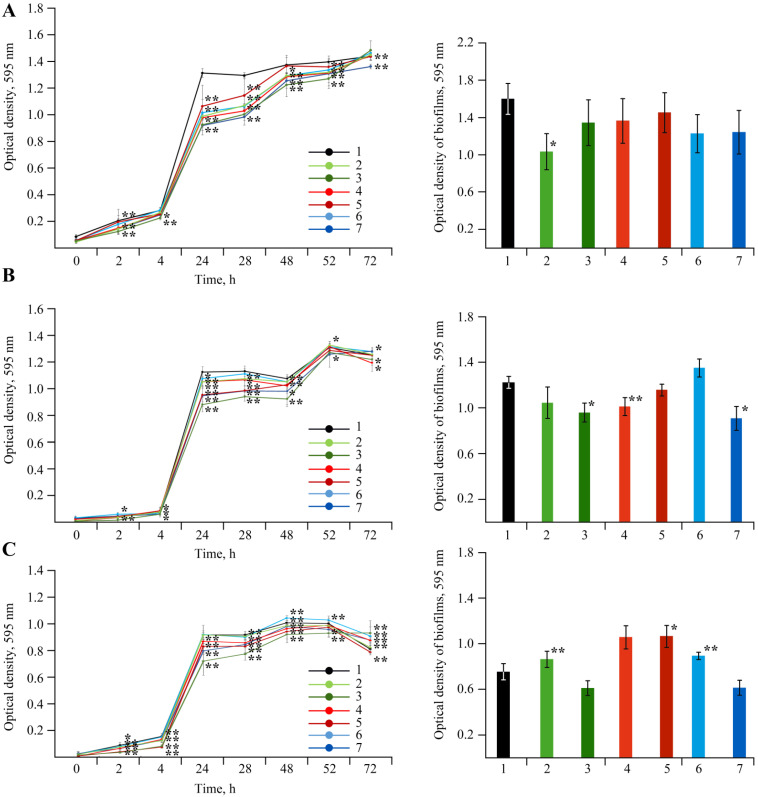
Effect of NC treatments with concentrations of Mn 0.000625% and 0.00625%^†^ in volume on growth (left graph) and biofilm formation (right graph) of *A. guillouiae* (**A**), *R. erythropolis* (**B**) and *P. oryzihabitans* (**C**); (1)—control, (2)—AG-Mn, (3)—AG-Mn^†^, (4)—AGS-Mn, (5)—AGS-Mn^†^, (6)—*κ*-CG-Mn, (7)—*κ*-CG-Mn^†^; * *p* ≤ 0.05 and ** *p* ≤ 0.01 compared to the control according to the Mann–Whitney test.

The effect of Mn-containing NCs on the growth of rhizosphere bacterial cultures of *A. guillouiae*, *R. erythropolis*, and *P. oryzihabitans* was evaluated in this study (Figure 17). It was shown that Mn NCs exhibited no bactericidal effect on these bacteria, and no precipitation zones were found around the wells with NCs.

## 3. Discussion

Analysis of the plant growth in various media revealed that the toxic effect of Mn accumulation in *S. tuberosum* L. culture manifested itself in enhanced dysfunctions of the plant organism (Figure 2 and Figure 3). The percentage of yellowed leaves was relatively low in the experiments without Mn sulfate in the nutrient medium, in the control (0.1 mol/L), and with 0.2–1 mol/L concentrations of the Mn sulfate salt, representing 13–15% of the total number of leaves on average. The maximum yellowing of leaves and their necrosis were observed at a concentration of 2 mol/L; the number of yellowed leaves reached 42% of their total number. Plants grown in a nutrient medium with a Mn sulfate salt concentration of 10 mol/L had no leaves on the 28th day of treatment. Eventually, the experiments allowed us to determine both the toxic effect of Mn and its optimal content in the culture medium of potatoes, as well as the required qualitative and quantitative physicochemical characteristics of this trace element in plant tissues.

Before carrying out experiments on plants and bacteria using NCs, the obtained NCs were characterized. The natural polysaccharides formed aggregate stable composites stabilized by a polymer shell. According to the data of TEM, EPR, and FTIR spectroscopy, the obtained nanoparticles were formed as Mn(OH)_2_×nH_2_O and were stabilized in the matrix on oxygen atoms and hydroxyl groups (Figure 5). It should be underlined that the formation of *κ*-CG-NC is somewhat different from that of AG and AGS polysaccharides. This is probably caused by the different structure of the polysaccharide chain and, as a consequence, by the specific packing of the polymer fiber, which is due to the fundamentally better solubility of AG owing to its hydrodynamic properties [21,22]. Therefore, this affects the amount of accumulated metal in the biopolymer volume as well as the size distribution of nanoparticles and anisotropy of the EPR signal.

EPR spectral characteristics of the signals (g-factor in the region of 2.0031(2), constant (A) 94(2) G of HFS) were almost identical to those observed in the spectra of biomaterials of control plants (Figure 7 and Figure 8). Comparison with the control revealed similar content of Mn in plant organs (Table 2), thus confirming that NCs were capable of acting as carriers of mineral fertilizers. Thus, a series of experiments demonstrated that Mn-containing NCs had no negative effect on the growth and development of potatoes. Moreover, NCs even stimulated development of the plants; AGS-Mn and *κ*-CG-Mn had the greatest effect. Their introduction into the potato cultivation medium promoted the development of an extensive root system.

The assessment of the stress state of plants under the action of the obtained NCs on biochemical parameters also produced interesting results. A significant increase in the activity of peroxidase was found in plants grown in a medium containing *κ*-CG-Mn in comparison with the control (Figure 9). The effect of other NCs on the enzyme activity was not revealed. The results showed that biotic stress can lead to a significant increase in the ROS content in the potato root tissues (Figure 10). In addition, an increase in the content of the primary products of lipid peroxidation—diene conjugates—was observed. In root tissues, treatment with all types of NCs contributed to a significant increase in the content of DCs. In leaf tissues, Mn NCs based on AG and sulfated AG also led to an increase in LPO products, but their amount was lower than in the case of potato infection with the pathogen (Figure 11). *κ*-CG-Mn NCs reduced the amount of DCs in both root and leaf tissues compared to infection without NC treatment. According to the content of MDA at the time point observed by us, there was indeed no surge in MDA during infection. Probably, at this moment, the MDA surge on infection had not yet occurred, since the disease of potato ring rot develops slowly; three days after infection with the pathogen, the potato had still not died, but the plants were already completely colonized by the phytopathogen *Cms*. In root tissues, AG-Mn NCs decreased the MDA content in both healthy and infected plants. κ-CG-Mn NCs reduced MDA production in the root tissues of healthy plants. At the same time, in leaves treated with Mn NCs based on AG and sulfated AG, LPO processes were significantly enhanced, judging by the increase in the MDA content (Figure 12). The obtained results indicate that AG-Mn NCs and Mn NCs based on sulfated AG did not have significant antioxidant activity. Some effect of reducing stress load was observed only under the influence of κ-CG-Mn NCs. This effect can be associated with the antioxidant activity of NCs [63,64,65,66], primarily due to single-electron transfer, the mechanism of which is discussed in detail in [63]. NCs with metal-containing nanoparticles, including Mn-oxide-containing nanocrystallites [65], exhibit antioxidant activity and, at the same time, are able to exhibit antibacterial activity against pathogenic bacteria.

It is important to note that the experiments demonstrated that all NCs exerted antibacterial effects against the phytopathogenic bacterium *Cms*. The maximum number of dead cells was detected in the cases of AGS-Mn and *κ*-CG-Mn at concentrations of 0.00625%. In addition, it was established that NCs altered the morphology of *Cms* bacterial cells (Table 3). After incubation of bacteria with NCs for 1 h, they lost their native shape and the cell surface became uneven, while Mn-containing nanoparticles were not detected. Probably, at the time of recording, the nanoparticles were not detected due to the fact that they had precipitated during the preparation of samples or dissolved in secondary metabolic processes, but had still had time to exert an antibacterial effect on *Cms*. It was revealed that after incubation with NCs, the cell walls of the bacteria were destroyed and the cell contents were released outside, which led to the death of the pathogenic bacteria (Figure 15 and Figure 16). The mechanism of the observed effect will be a subject of a separate study. It should be mentioned that a similar process took place with selenium-containing NCs and may be associated with a violation of the cell membrane potential caused by the attachment of Mn-containing nanoparticles to its surface [67].

Experiments on the effect of the NCs on the natural soil microbiome also gave good results. It was established that on the first day of the co-incubation of *A. guillouiae*, *P. oryzihabitans*, and *R. erythropolis* with NCs, cell growth was only slightly inhibited as compared to the control. Furthermore, this effect leveled out over time. *R. erythropolis* is a Gram-positive bacterium, but *A. guillouiae* and *P. oryzihabitans* are Gram-negative bacteria; thus the difference in the structure of the bacterial cell wall was also a reason for testing the effect of NCs on these strains. It is known that in most cases Gram-negative bacteria are more resistant to stress factors than Gram-positive ones [68]. The high resistance of Gram-negative microorganisms to NCs is provided by the presence of two membranes in the cell wall, outer and inner. Due to this structure of the cell wall, Gram-negative microorganisms demonstrate greater resistance even to antibiotics [69]. Therefore, no effect of NCs on the viability of *A. guillouiae* and *P. oryzihabitans* was found. In turn, *R. erythropolis* in the systematics of microorganisms belongs to the order *Actinomycetales*, the family Mycobacteriaceae, and the genus *Actinobacteria*; and *Cms* belongs to the genus of rod-shaped actinobacteria *Corynebacterium* from the family Microbacteriaceae of the order *Actinomycetales*. Thus, they are similar microorganisms and belong the same order *Actinomycetales* of Gram-positive bacteria. However, *R. erythropolis* contains mycolic acids in its cell wall [70,71]. Mycolic acids are long-chain branched fatty acids [72]. They are found only in the cell wall of *Mycobacteria*, including the cell wall of the causative agent of tuberculosis *Mycobacterium tuberculosis* [73]. Mycolic acids ensure the resistance of bacterial cells to various harsh stress factors (incubation in acids, alkalis, boiling). These acids have low reactivity, which makes the surface of mycobacteria waxy and extremely hydrophobic [74]. According to multiple studies, mycolic acids ensure the resistance of *R. erythropolis* to various stress factors and pollutants, such as oil products and heavy metals [75,76,77,78,79], and also allow survival on very poor nutrient media [80]. We assume that the presence of a negative effect of NCs on the viability of *Cms* is associated with the absence of mycolic acids in the cell wall of this bacterium. Thus, resistance to the influence of NCs and the viability of soil bacteria are apparently due to the natural features of bacteria cell walls.

## 4. Materials and Methods

### 4.1. Synthesis of Manganese-Containing NCs

The AG was obtained from a polysaccharide of Siberian larch, *Larix sibirica* Ledeb. (Wood Chemistry Ltd., Irkutsk, Russia) [22]. It was additionally purified of impurities and flavonoids by passing it through a polyamide column. The AGS was obtained by modification of the original AG according to the patent [81], and *κ*-CG was obtained from CP Celko Comp. (Lille Skensved, Denmark). Previously, we studied the effect of original polysaccharides on potatoes (*Solanum tuberosum* L.) and bacteria, in which the polysaccharides did not have a negative effect on plants and even stimulated the growth of *Cms* colonies and rhizosphere bacteria [82,83,84,85]. NCs were synthesized according to the following procedures:

AG-Mn NC: AG (2 g, 18 kDa) was dissolved in H_2_O (5 mL), and MnSO_4_×5H_2_O (0.4 g) in H_2_O (3 mL) was added to the solution. Afterwards, NH_4_OH (0.1 mL) and hydrazine (0.2 mL) were added upon stirring with a magnetic stirrer. After 3 h of stirring, the reaction mixture was precipitated in alcohol and dried. Washing with alcohol gave 1.68 g of AG-Mn NC.

AGS-Mn NC: AGS (2 g, 22 kDa) was dissolved in H_2_O (6 mL), and MnSO_4_×5H_2_O (0.3 g) in H_2_O (2 mL) was added to the solution. Afterwards, NH_4_OH (0.2 mL) was added upon stirring with a magnetic stirrer. After 4 h, the reaction mixture was precipitated in alcohol and dried. Washing with alcohol gave 1.8 g of AGS-Mn NC.

*κ*-CG-Mn NC: *κ*-CG (3 g, 1100 kDa) was kept under stirring in H_2_O (150 mL) upon heating at 50 °C to a homogeneous medium. Then, MnSO_4_×5H_2_O (0.69 g) in H_2_O (3 mL) and NH_4_OH (0.3 mL) were added. After 24 h, the reaction product was precipitated into alcohol. Thorough washing of the precipitate with alcohol gave 2.4 g of *κ*-CG-Mn NC.

The obtained NCs were fine brown or light brown colored powders with the color intensity being dependent on the mass content of Mn in the sample. The average mass values of Mn in NCs AG-Mn, AGS-Mn, and *κ*-CG-Mn were 5.2, 4.8, and 20.3 wt%, respectively.

### 4.2. Physicochemical Measurements

To study the morphology of Mn-containing NC films, the obtained AG-Mn, AGS-Mn, and *κ*-CG-Mn were dissolved in water. Then, their water solutions were applied to grids with formvar supports and dried. The prepared samples of NC films were examined using a Leo 906 E (Carl Zeiss, Jena, Germany) transmission electron microscope (TEM) at an accelerating voltage of 80 kV. Micrographs were taken with a MegaView II camera and processed using Mega Vision software.

Elemental analysis of the obtained NCs was performed on a Flash EA 1112 CHNS-O/MAS 200 analyzer, and the percentage of Mn in the NCs was determined using the energy-dispersive X-ray spectroscopy function built into the scanning electron microscope TM 3000 chamber (Hitachi, Tokyo, Japan) equipped with a SDD XFlash4304-H detector for imaging, where they were subjected to electron impact. Atoms of the sample were excited via electron beam and thus emitted X-rays of wavelengths characteristic of each chemical element. Analyzing the energy spectrum of X-ray emissions, we assessed the sample qualitative and quantitative composition. The number of repetitions for each sample was five, as well as five measurement areas in each sample. The obtained percentages are presented in Table 4.

Molecular weight was determined using gel permeation chromatography on a column filled with Sephadex G-100 (24 × 350 mm). Dextran (20, 40, 100, and 2000 kDa) and D-galactose standards were used for calibration. An isotonic solution was used as an eluent.

The FTIR spectra were recorded on a Bruker Vertex 70 FT-IR instrument in KBr pellets.

The percentage of Mn in the plant material was determined by atomic absorption analysis using a TM 3000 scanning electron microscope (Hitachi, Tokyo, Japan) equipped with a SDD XFlash 4304-H detector. Elemental analysis was carried out on the tissues of roots, stems, and leaves for each series of plants grown on a nutrient medium with different concentrations of Mn salt. The biological material was dried in a SSh-80-02 SPU dry-heat oven at 60 °C for 16 h and then was calcined in a LOIF LF-5/11-G1 muffle furnace at 350 °C for 1 h, the resulting ash was analyzed for metal content. The number of repetitions for each sample was five, as well as five measurement areas in each sample.

The EPR spectra were recorded on a FT Bruker ELEXSYS E-580 spectrometer (X-band 9.7 GHz). CW EPR spectra were recorded under the following conditions (in quartz ampoules with a diameter of 3 mm): modulation amplitude 1–10 Gs, frequency modulation 100 kHz, time constant 0.02 s, conversion time 0.06 s, microwave power 0.6325 mW, average number of scans 20, receiver gain 50 dB at room temperature.

### 4.3. Plant Material

In vitro plants of the Lukyanovskii potato variety, which is susceptible to biotic stresses, were used in the study [86]. Potato plants were cultivated under factor-static conditions on the Murashige–Skoog growth medium (Sigma–Aldrich, Inc., St. Louis, MO, USA), where Mn was added in the form of Mn sulfate crystallohydrate MnSO_4_×5H_2_O.

The concentrations of Mn sulfate varied from 24.1 g/L (mass content for control according to the prescription of the Murashige–Skoog liquid nutrient medium) to a 100-fold excess from the norm. Such concentrations were used to study the toxic effects of Mn and its accumulation in plant organs in a medium with an increased metal content, as well as in the medium with a complete absence of Mn sulfate salt. Nutrient media without Mn sulfate and with 2, 5, 10, 20, and 100-fold excesses of the Mn sulfate salt were used (control concentration was 0.1 mol/L).

To study the effect of natural polysaccharide-based NCs on the growth and productivity of potatoes, the plants were cultivated under factor-static conditions in the Murashige–Skoog liquid nutrient medium, with MnSO_4_×5H_2_O replaced by NCs. The amount of the composite was determined according the required content of Mn in the medium according to the prescription based on the mass fraction of the metal in the NC (based on the mass of elemental Mn).

The potato cuttings were planted to the internode depth in agar nutrient medium and cultivated for 28 days under factor-static conditions at 24–25 °C, illumination of 5–6 Klx, and a photoperiod of 16 h, while periodically measuring the length of the plants and counting the number of leaves. At the end of the experiment, the biomass of the aboveground part and the biomass of the roots were determined and the physicochemical properties of the plant material were analyzed. Independent experiments were repeated three times; in each experiment, ten plants were grown. For the physicochemical study, three plants from each experiment were used.

The results were subjected to statistical analysis using the MS Excel statistic add-in software package, and comparisons with the control were evaluated according to the Mann–Whitney test.

### 4.4. Stress Resistance Experiments

To identify areas of production of ROS, the plants were infected with *Cms*, kept for four days, then treated with NCs; after 1 h of co-incubation, samples were prepared for analysis. Plant root tissue samples were incubated for 30 min with 5 μM CellROX Deep Red Reagent (abs/em 644/665 nm) (Thermo Fisher Scientific Inc., Waltham, MA, USA) dissolved in phosphate buffer. Then the tissue was fixed with 2% paraformaldehyde for 15 min. The obtained preparations were embedded in ProLong Gold antifade reagent (Thermo Fisher Scientific Inc., Waltham, MA, USA) and examined with a CLSM 710 confocal microscope (Carl Zeiss, Jena, Germany). The study used 405 and 561 nm lasers and filters Ch1 410–522.

The content of ROS in potato roots was determined spectrophotometrically using a xylenol orange dye [87]. The determination of the primary products of LPO and DC in the tissues of potato plants was carried out according to the standard method using hexane and isopropanol 30 and 60 min after the addition of the NC solution into the potato growth medium in vitro [88,89]. The concentration of MDA was determined by the method using 20% trichloroacetic acid and 0.5% thiobarbituric acid solution [90]. Peroxidase activity in potato tissues was determined according to the Boyarkin method [47].

The results were subjected to statistical analysis using the MS Excel statistic add-in software package, and comparisons with the control were evaluated according to the Mann–Whitney test.

### 4.5. Bactericidal and Bacteriostatic Effect of the NCs

The strain Ac-1405 of gram-positive bacterium *Cms* causing potato ring rot was used in this study. It was obtained from the All-Russian collection of microorganisms (GK Skryabin Institute of Biochemistry and Physiology of Microorganisms, Russian Academy of Sciences, Pushchino, Moscow Region); the strain was cultivated on GPY medium [91] containing yeast extract 5 g/L (Sigma-Aldrich, Inc., St. Louis, MO, USA), glucose 5 g/L (Diaem, Moscow, Russia), peptone 10 g/L (Sigma-Aldrich, Inc., St. Louis, MO, USA), NaCl 5 g/L (NevaReaktiv, Saint Petersburg, Russia), and agar 7 g/L (Diaem, Moscow, Russia). To maintain the culture, the tubes with slant agar were placed in a thermostat at 25 °C. For the experiment, the bacterial colony was transferred from a solid nutrient medium to a liquid one and grown for 2 days.

A 0.05% aqueous solution of NCs and their precursors were added to flasks with a bacterial suspension (optical density D = 0.9). The solutions of all compounds were preliminarily subjected to cold sterilization (syringe nozzle “Minisart NML”, pore size 0.22 µm). The concentration of the active substance was equal for all compounds: 0.000625% and 0.00625% (based on the mass of Mn in the precursor). When choosing the effective concentrations, we were guided by the concentrations used for selenium-containing NCs based on polysaccharides; in previous experiments, NCs were used at a final concentration of 0.000625% selenium in a solution [6,84,92]. Similarly, in this work, NCs were used at a final concentration of 0.000625%Mn in a solution, as well as in a concentration 10 times higher, of 0.00625% Mn, to evaluate the effectiveness of antibacterial activity and the effect of Mn if a higher concentration is formed in the medium. To study the bacteriostatic activity of NCs against bacteria, a liquid culture of microorganisms was grown in the dark at 26 °C under aerated conditions (80 rpm) in flasks containing a GPY nutrient medium (pH 7.2). After the addition of the composites, the optical density of the suspension was measured at 595 nm on a Bio-Rad spectrophotometer model 680 (Bio-Rad Laboratories, Inc., Hercules, CA, USA) immediately and after 2, 4, 24, 28, 48, 48, 52, and 72 h of co-incubation.

The bactericidal effect of NCs was determined using the circle method (agar diffusion method) [93].

The effect of NCs on bacterial biofilm formation was investigated using the tablet method. In the experiments, the dead cells were stained with propidium iodide (PI) dye at a final concentration of 7.5 μM (Biotium, Fremont, CA, USA) [94]; during the experiment, bacterial cells were incubated with NCs for 24 h. The bacteria were studied using an AxioObserver inverted fluorescence microscope Z1 (Carl Zeiss, Jena, Germany). Micrographs were taken with an AxioCam MRm3 camera and processed using AxioVision Rel.4.8.2 software.

After incubation with NCs for 1 day, *Cms* bacteria were fixed in a 2.5% solution of glutaraldehyde on a 0.1 M phosphate buffer (pH 7.4) for 1 h, washed with the same buffer, additionally fixed with a 2% solution of osmium tetroxide (1–3 h), and poured into epoxy resin. Slices were made on an Ultracut R ultramicrotome (Leica Microsystems GmbH, Wetzlar, Germany). After staining in lead citrate, they were examined using a Leo 906 E TEM.

The results were subjected to statistical analysis using the MS Excel statistic add-in software package, and comparisons with control were evaluated according to the Mann–Whitney test.

### 4.6. Experiments with Soil Bacteria

As soil microorganisms, we used bacteria isolated from the rhizosphere of plants growing in the oil-contaminated territory of Tyret village (Zalarinsky District, Irkutsk Region, Russia). After the isolation of the bacteria, their morphological, cultural, physiological, and biochemical properties were studied. In addition, the 16S rRNA gene was sequenced to determine the species composition [95].

The *Acinetobacter guillouiae* strain was isolated from *Elytrigia repens* wheatgrass rhizosphere. The cells are short rods, Gram-negatively stained. The bacteria (aerobes, oxidase and catalase-positive) form colonies up to 1.2 mm in size. The colonies are beige, round, shiny, slightly convex, and homogeneous, with a soft consistency and an even edge.

The *Rhodococcusery thropolis* strain was also isolated from the rhizosphere of *E. repens* quack grass. At an early stage of development, the culture is a rudimentary mycelium, which later breaks down into fragments. The fragments then turn into rods and then into cocci. The bacteria are Gram-positive. Their colonies are medium, about 1.5–2.5 mm in size. They are cream-colored, matte, pasty, and convex, with a rhizoid edge and a rough structure. They are aerobes, oxidase-negative, and have catalase activity.

The *Pseudomonas oryzihabitans* strain was isolated from *Carex hancockiana* Maxim. sedge rhizosphere. The cells are single or paired straight rods, Gram-negatively stained. The bacteria (aerobes, catalase and oxidase-positive, capable of pigmentation), when cultivated on solid media, form yellow, smooth, shiny, round with smooth edges, convex, homogeneous, soft consistency, slimy colonies [96].

To clarify the species composition of pure cultures, the nucleotide sequence of 16S rRNA gene was analyzed, which showed the following nucleotide sequences in size: 1136 bp (98%), 1429 bp (99%), and 1098 bp (98%) that corresponded to *Rhodococcus* spp., *Pseudomonas* spp. and *Acinetobacter* spp., respectively.

The bacteria were cultivated in the dark for one day on a solid medium consisting of agar, enzymatic hydrolyzate of beef meat and on a liquid nutrient medium of a similar composition. The possible bactericidal effect of NCs was determined using the circle method (the method of diffusion in agar) [93]. The effect of NCs was evaluated by the width of the inhibition zone around the wells on the media plate.

To study the bacteriostatic activity of NCs against bacteria, a liquid culture of microorganisms was grown in the dark at 26 °C under aerated conditions (80 rpm) in flasks containing a nutrient medium. After the addition of NCs, the optical density of the suspension was measured at 595 nm on a Bio-Rad plan-table spectrophotometer immediately and after 2, 4, 24, 28, 48, 52, and 72 h of co-incubation.

The experiments were carried out in three independent biological replicates. The data obtained were subjected to statistical analysis using the MS Excel statistic add-in software package and evaluated according to the Mann–Whitney test.

## 5. Conclusions

According to the results obtained, it can be concluded that the studied NCs are safe for soil microbiome species. We were able to show the prospects for the development of multi-purpose organomineral fertilizers for plant protection and targeted delivery of microelements based on biocompatible, safe, and easily biodegradable nanomaterials. In addition, polysaccharide-based fertilizers will ensure a prolonged effect and will be resistant to the leaching of microelements from different types of soil.

Finally, the general toxicological effect of Mn and its qualitative and quantitative physicochemical characteristics were also studied. The optimum content of this trace element in the culture medium of potatoes was determined. The results obtained are important for better understanding the mechanisms of potato survival on saline soils and in private subsidiary farms in the immediate vicinity of industrial enterprises, as well as for the evaluation of plants’ ability to accumulate metals. In the present work, we have used Mn-containing NCs based on natural polysaccharide matrices as multi-purpose trophic low-dose micronutrients. The Mn NCs can be employed as safe and biodegradable carriers of mineral trace elements possessing biologically active properties for plant protection. As compared to the control, potato plants grown in media with Mn NCs demonstrated similar results in terms of elemental analysis and EPR characteristics, and were superior in terms of biometric indicators. The observed effect is probably due to the synergetic bioactive action of the polysaccharides and nano-sized particles, which stimulates the growth of plant biomass. It also should be noted that the obtained Mn NCs have a pronounced antibacterial effect against the phytopathogenic bacterium *Cms* and the formation of its bacterial biofilms, while being safe for soil microflora species. Thus, the use of such metal-containing NCs opens up new frontiers in the design of universal micronutrient fertilizers and their application in biology, gardening, and agriculture.

## Figures and Tables

**Table 1:** The accumulation of Mn (wt% ± SD) in organs of potato plants grown in media with different concentrations of Mn sulfate.

Mn Sulfate, mol/L	Roots	Stems	Leaves
0	0.02 ± 0.01 *	0.04 ± 0.01 *	0.14 ± 0.02
0.1 (control)	0.11 ± 0.02	0.18 ± 0.01	0.16 ± 0.04
0.2	0.17 ± 0.04	0.21 ± 0.04	0.22 ± 0.01 *
0.5	0.54 ± 0.01 *	0.29 ± 0.08 *	0.20 ± 0.08
1.0	0.79 ± 0.09 *	0.88 ± 0.28 *	0.34 ± 0.08 *
2.0	0.97 ± 0.08 *	1.10 ± 0.13 *	0.74 ± 0.15 *
10.0	1.50 ± 0.25 *	5.34 ± 0.59 *	3.82 ± 0.17 *

* *p* ≤ 0.01 compared to the control according to the Mann–Whitney test.

**Table 2:** The accumulation of Mn (wt% ± SD) in plants grown in the control medium and in a medium with NCs.

Treatment	Roots	Stems	Leaves
Control	0.17 ± 0.03	0.19 ± 0.04	0.20 ± 0.13
AG-Mn	0.28 ± 0.10 *	0.27 ± 0.01 *	0.24 ± 0.20
AGS-Mn	0.05 ± 0.04 *	0.16 ± 0.11	0.17 ± 0.13
*κ*-CG-Mn	0.29 ± 0.11 *	0.38 ± 0.13 *	0.24 ± 0.11

* *p* ≤ 0.01 compared to the control according to the Mann–Whitney test.

**Table 3:** Effect of NC treatments on morphological parameters of *Cms* bacterium (length, width, and dead cell count) with concentrations of Mn 0.000625% and 0.00625%^†^ in volume.

Treatment	Length, µm	Width, µm	Dead Cell Count, %
Control	3.13 ± 0.08	0.56 ± 0.02	0.34 ± 0.01
AG-Mn	2.12 ± 0.07 *	0.49 ± 0.01 *	2.35 ± 0.46 *
AG-Mn^†^	2.07 ± 0.08 *	0.65 ± 0.19	5.55 ± 0.40 *
AGS-Mn	2.31 ± 0.09 *	0.53 ± 0.01	3.69 ± 0.54 *
AGS-Mn^†^	2.53 ± 0.34 *	0.77 ± 0.01 *	24.77 ± 1.70 *
*κ*-CG-Mn	2.41 ± 0.63 *	0.62 ± 0.04 *	3.07 ± 0.66
*κ*-CG-Mn^†^	2.62 ± 0.17 *	0.71 ± 0.24 *	13.68 ± 4.82 *

* *p* ≤ 0.01 compared to the control according to the Mann–Whitney test.

**Table 4:** Elemental percentages (wt%) of the obtained NCs.

Nanocomposite	C	H	S	Mn
AG-Mn	38.5	5.8	0.0	5.2
AGS-Mn	28.7	5.8	7.1	4.8
*κ*-CG-Mn	26.8	5.3	2.5	20.3

## Data Availability

Data are contained within the article.
